# Two Cases of Pachydermodactyly Presenting as Polyarthritis

**DOI:** 10.1155/2018/9835279

**Published:** 2018-01-17

**Authors:** Roxana Mititelu, Sarah Finch, Paul Dancey, Ian Landells

**Affiliations:** ^1^Department of Dermatology, McGill University Health Centre, Montreal, QC, Canada; ^2^Department of Pathology, Memorial University, St. John's, NL, Canada; ^3^Department of Rheumatology, Memorial University, St. John's, NL, Canada; ^4^Department of Dermatology, Memorial University, St. John's, NL, Canada

## Abstract

Pachydermodactyly is characterized by asymptomatic, progressive swelling of the lateral aspects of the 2nd to 4th finger along the proximal interphalangeal (PIP) joint without involving the joint itself. We present 2 interesting cases of patients with periarticular swelling who were initially diagnosed and treated as juvenile idiopathic arthritis (JIA) with subsequent clinical and pathology confirmation of pachydermodactyly. These cases emphasize the importance of considering pachydermodactyly in young patients with development of periarticular swelling and no joint involvement.

## 1. Introduction

Pachydermodactyly is a rare, benign, acquired digital fibromatosis characterized by asymptomatic, progressive swelling of periarticular soft tissues of the fingers without joint involvement. The diagnosis is usually made clinically; however, histopathology will demonstrate coarse collagen bundles and dermal mucin deposition. The etiology is believed to be mechanical trauma in most cases. Treatment is often not indicated given its benign prognosis.

## 2. Case Reports

### 2.1. Case  1

**Figure 1:**
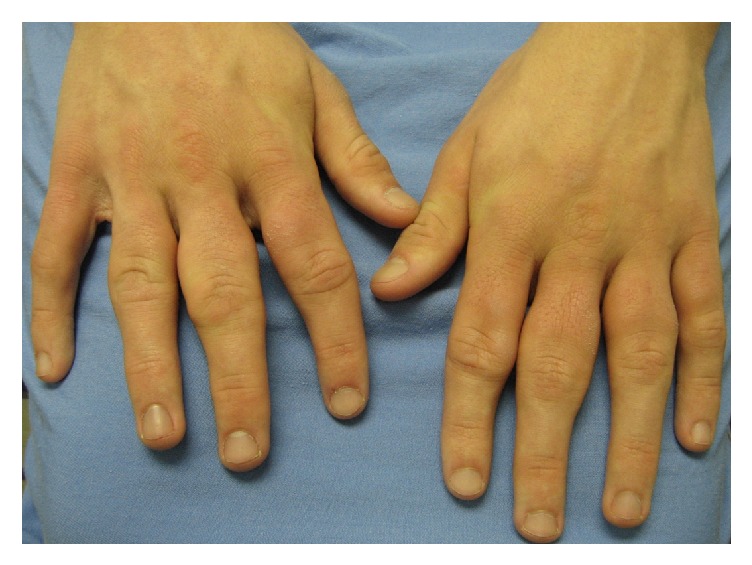
Periarticular nodules involving mainly the PIP joints of the 2nd to 4th finger of both hands.

A 17-year-old young man was seen in the pediatric rheumatology clinic for investigation of presumed JIA polyarthritis affecting his hands. The patient described the gradual appearance of discrete “swellings” of his fingers beginning 5 years earlier, without pain, or significant physical limitations. He reported mild morning stiffness of short duration and fatigue in the hands after prolonged writing. He was otherwise well, and a review of systems was unremarkable. Prior to the rheumatology assessment, he was seen by his pediatrician who diagnosed JIA and initiated NSAID treatment. On physical exam, a nodular swelling was noted adjacent to the PIP and to a lesser extent the metacarpophalangeal (MCP) joints of both hands (Figure 1). There was minimal tenderness on palpation and range of motion was preserved.

The radiograph of the hands revealed soft tissue swelling concordant with the clinical presentation, but no joint space narrowing or erosions. Magnetic Resonance Imaging (MRI) with gadolinium of the hands did not reveal signs of synovitis. Complete blood count (CBC), antinuclear antibody (ANA), rheumatoid factor (RF), and inflammatory markers were all normal.

**Figure 2:**
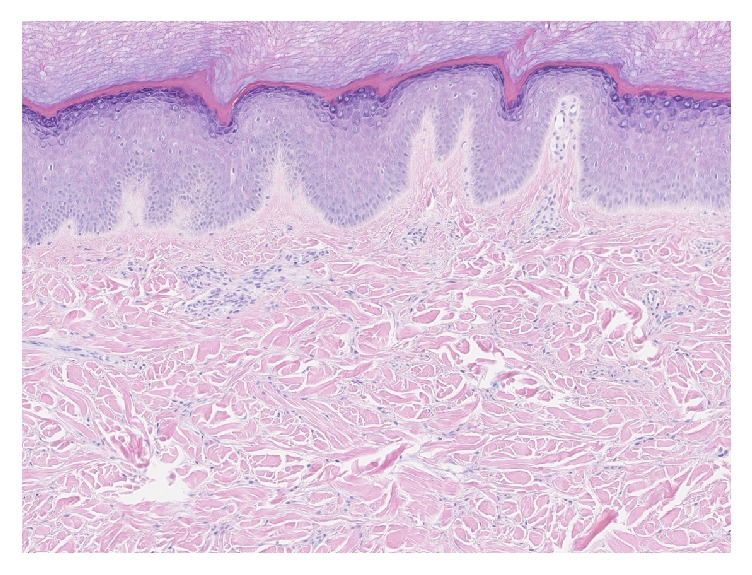
Histological examination revealed coarse dermal collagen bundles with perieccrine collagen deposition, increased fibroblasts, reduction in elastic fibers, and scant perivascular lymphocytic inflammation (hematoxylin-eosin stain; original magnification ×10).

**Figure 3:**
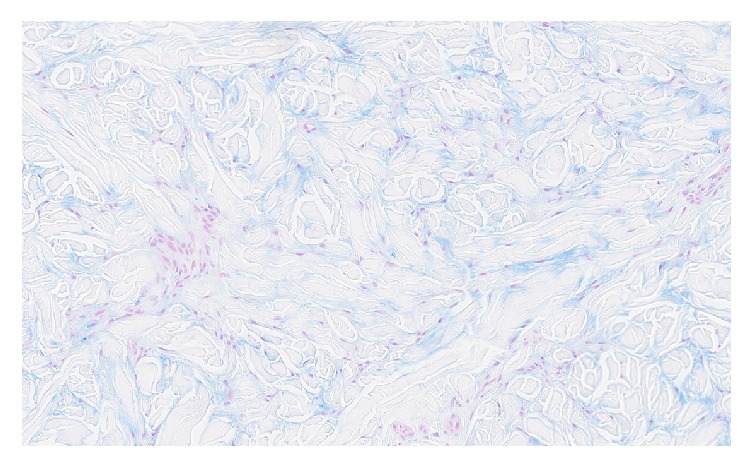
Alcian blue stain showing increased dermal mucin deposition (Alcian blue; original magnification ×10).

As the presentation was not typical for JIA, a biopsy of a nodule was performed to clarify the diagnosis. Histological examination revealed coarse dermal collagen bundles with perieccrine collagen deposition, increased fibroblasts, reduction in elastic fibers, and scant perivascular lymphocytic inflammation (Figure 2). Furthermore, alcian blue and colloidal iron confirmed increased dermal mucin deposition (Figure 3).

A diagnosis of pachydermodactyly was provided and dermatology was consulted for further management. During the subsequent 4 years, the patient remained well with no change in the condition. As the patient was concerned about the appearance of his hands, a trial of hydroxychloroquine was then initiated, but discontinued after 1 month due to a side effect of vertigo. One year later, he remained well with no change in the condition.

### 2.2. Case  2

**Figure 4:**
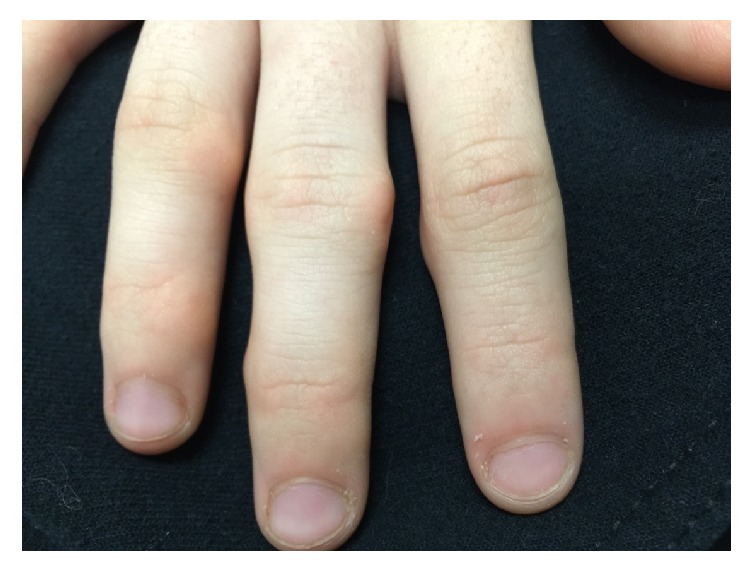
Periarticular nodules of the PIP and DIP joints.

A 14-year-old girl was referred to pediatric rheumatology for investigation of a one-year history of “joint swelling” of the fingers in her right hand. She described some mild discomfort when opening and closing her hand, but no morning stiffness or other physical limitations. She had occasional arthralgia of her knees with activity, but was otherwise well, and a review of systems was unremarkable. There was a significant family history of rheumatoid arthritis. Physical exam revealed a nodular prominence, with mild tenderness, in the area of her PIP and DIP joints of the right hand (Figure 4). She had limited active but normal passive flexion of the fingers. The remainder of the musculoskeletal exam and general physical was normal.

Investigations including CBC, ANA, RF, thyroid testing, and inflammatory markers were all normal. The radiograph of the right hand revealed soft tissue swelling, but no joint space narrowing or erosions. A subsequent MRI with gadolinium of the hands was of poor quality due to motion artifact, but was reported as showing possible synovitis, resulting in a preliminary diagnosis of polyarticular JIA. The patient was then started on a NSAID and methotrexate therapy.

**Figure 5:**
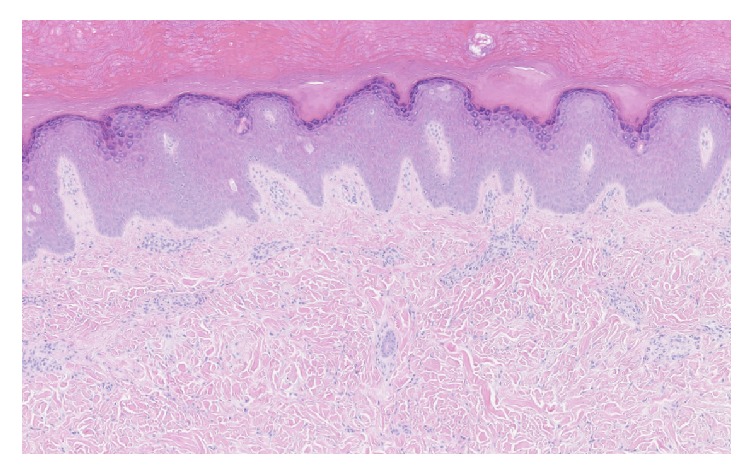
Histology examination showed increased collagen bundles, increased fibroblasts, reduction in elastic fibers, and scant perivascular lymphocytic inflammation (hematoxylin-eosin stain; original magnification ×4).

**Figure 6:**
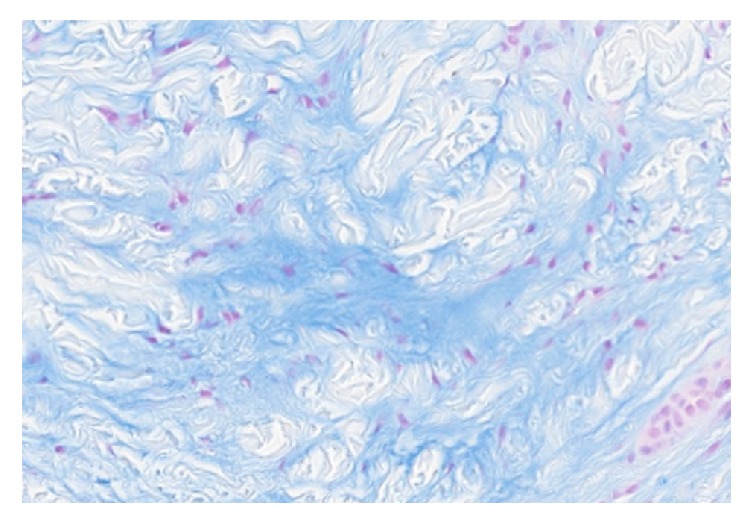
Alcian blue stain showed increased mucin deposition in the full thickness of the dermis (Alcian blue; original magnification ×10).

Despite six months of treatment, she reported no change in her symptoms or the appearance of her hand, prompting reconsideration of the arthritis diagnosis. Subsequently, a biopsy of one of the nodules was performed. Histology showed increased mucin deposition in the full thickness of the dermis, strongly positive for alcian blue and colloidal iron staining (Figures 5 and 6). There was also evidence of increased collagen bundles, increased fibroblasts, reduction in elastic fibers, and scant perivascular lymphocytic inflammation. The patient was then referred to dermatology for the management of pachydermodactyly. Given the patient's concern about the appearance of her hands, she was offered treatment with hydroxychloroquine. After 6 months of therapy, her lesions were showing mild improvement but nevertheless persisting. The patient chose to stop further treatment.

## 3. Discussion

Pachydermodactyly is an uncommon type of digital fibromatosis. Typically, it presents in adolescent boys as swelling of the soft tissue involving the lateral aspects of the PIP joints particularly of the second to the fourth fingers. Involvement of other joints including the DIP and the MCP, consistent with our patients' clinical appearance, has also been described [1]. In certain cases, the disorder is hypothesized to be related to mechanical trauma. In a review of pachydermodactyly, mechanical trauma was found to be a precipitant factor in 44% of patients. Activities which have been implicated include martial arts, rock climbing, and repetitive labour [1]. The histopathology is characterized by hyperkeratosis and acanthosis of the epidermis [2]. The dermis is characterized by coarse collagen bundles with an increase in fibroblasts [2]. There can also be mucin deposition of varying degrees in the interstitium and decreased elastic fibers [2].

The treatment of this condition is not well-defined and few studies examining treatment options have been published. Elimination of mechanical stimulation with occupational therapy has been shown to be helpful [1]. Oral tranilast, intralesional triamcinolone injection, and surgical excision have been used with benefit in some cases [3–5]. Interestingly, in other forms of fibromatoses, such as Dupuytren's contracture, injectable collagenase has been shown to be promising with a response rate of 45–65% [6].

Here, we have described 2 additional cases, who were initially referred for possible JIA prior to clinical and histopathological confirmation of pachydermodactyly. JIA is also a relatively rare condition in which rheumatoid nodules may develop over MCP and PIP joints in polyarticular disease. A key distinguishing feature is joint involvement in JIA. Other differential diagnoses include self-healing juvenile cutaneous mucinosis and acral persistent papular mucinosis. Self-healing juvenile cutaneous mucinosis is less likely given the lesions persisted in our patients. While acral persistent papular mucinosis has similar histopathologic findings, the primary skin lesions are papules rather than the nodules seen in our patients. While milder cases of pachydermodactyly can be observed and patients can be counselled to avoid any possible aggravating factors, both patients were concerned about the appearance of their hands. Consequently, we proposed treatment with hydroxychloroquine given its anti-inflammatory and antiproliferative actions. As the pathogenesis of pachydermodactyly is driven by fibroblastic proliferation and resultant collagen deposition, we feel there is some rationale to this approach [1]. One of our patients showed some improvement of the lesions during a 6-month course of hydroxychloroquine before stopping. Ideally a longer follow-up will be needed to determine the outcome and possible effectiveness of this treatment approach.

## 4. Conclusion

Pachydermodactyly is a rare condition which can mimic the appearance of arthritis. Careful attention to the physical exam, particularly to distinguish between an intra-articular and a cutaneous process, is important to facilitate arrival at the correct diagnosis and avoid potentially unnecessary investigations. More research is needed to determine the optimal treatment choice for this rare condition.
